# Supplementary material for: A comparative analysis of infection and mortality in reassessing africa’s COVID-19 dynamic using time-varying tests
Source: Commun Med (Lond). 2026 Feb 6;6:92. doi: 10.1038/s43856-025-01343-2 (PMC12886758; doi:10.1038/s43856-025-01343-2)

Supplementary Table 1: Countries by WHO region

The table lists the 89 countries included in the primary database, which follows a strict selection rule (requiring at least one observation every two weeks between May 1, 2020, and January 1, 2022). It also shows the countries that are added to the secondary database when this selection rule is relaxed. For AFR, 22 countries are therefore included in the secondary database, that is, about half of the African countries.

**WHO Region**	**Primary database**	**Secondary database**
AFR	Botswana, Cote d'Ivoire, Senegal, South Africa, Togo, Zambia, Zimbabwe, Nigeria, Namibia, Rwanda	Equatorial Guinea, Ethiopia, Gabon, Gambia, Ghana, Kenya, Madagascar, Malawi, Mauritania, Mozambique, Uganda, South Sudan
AMR	Argentina, Bolivia, Chile, Colombia, Costa Rica, Ecuador, Guatemala, Jamaica, Paraguay, Peru, Uruguay, Panama, Mexico, United States, Canada, Puerto Rico, United States, Virgin Islands	Antigua and Barbuda, Bahamas, Belize, Brazil, Cuba, Dominican Republic, El Salvador, Haiti, Saint Kitts and Nevis, Saint Vincent and the Grenadines Suriname, Trinidad and Tobago, Anguilla, Aruba, Curacao
EUR	Albania, Armenia, Austria, Bosnia and Herzegovina, Belgium,Bulgaria, Croatia, Cyprus, Czechia, Denmark, Estonia, Finland, France, Georgia, Germany, Greece, Ireland, Israel, Italy, Latvia, Lithuania, Luxembourg, Malta, Netherlands, Norway, Poland, Portugal, Romania, Russia, Serbia, Slovakia, Slovenia, Spain, Sweden, Switzerland, Turkey, Ukraine, United Kingdom, Liechtenstein	Kazakhstan, Azerbaijan, Belarus, North Macedonia, Andorra, Kosovo
SEAR	Bangladesh, India, Maldives, Nepal, Sri Lanka, Thailand	Bhutan, Myanmar, Indonesia, North Korea
EMR	Bahrain, Iran, Iraq, Kuwait, Morocco, Pakistan, Saudi Arabia, United Arab Emirates	Jordan, Lebanon, Qatar, Tunisia
WPR	Australia, Japan, Malaysia, New Zealand, Philippines, South Korea, Guam	China, Brunei, Cambodia, Fiji, Laos, Marshall Islands, Mongolia, Vietnam, Northern Mariana Islands

Supplementary Method 1: Mathematical Derivation of the Acceleration Index

The purpose of this appendix is to present the mathematical derivation of the acceleration index used in our analysis of African country data, assuming for convenience that time t is positive and continuous. In addition, suppose that there are n groups (e.g. spatial as for the present paper) indexed by i and such that $P_i(t) = \alpha_i \pi_i(t) \geq 0$ and $D_i(t) = \gamma_i \delta_i(t) \geq 0$ are the numbers of positive cases and tests *cumulated up to date t*. Note that the scale (i.e. level) parameters $\alpha_i > 0$ and $\gamma_i > 0$ are group (e.g. country or region)-specific but time-invariant, to capture the fact that some groups may be more or less tested, or more or less susceptible to the virus, than others. The functions $\pi_i(t)$ and $\delta_i(t)$, on the other hand, capture the fact that the *dynamics* of cases and tests may also be group-specific. Using this notation, it follows that the time derivatives $P_i'(t) = \alpha_i \pi_i'(t)$ and $D_i'(t) = \gamma_i \delta_i'(t)$ - which are assumed to be non-negative - represent the instantaneous new cases and tests, that are here the theoretical analog of what we denote p_t and d_t in the data analysis.

While $P_i(t)$ and $D_i(t)$ are by definition non-decreasing over time, the cumulative positivity rate for each group i, defined - when $D_i(t) > 0$ - as $P_i(t)/D_i(t)$, is strictly increasing over time if and only if its derivative with respect to time t is strictly positive, i.e.

$$\frac{d(P_i(t)/D_i(t))}{dt} = \frac{P_i(t)}{D_i(t)} \left[\frac{P_i'(t)}{P_i(t)} - \frac{D_i'(t)}{D_i(t)} \right] > 0$$

or, equivalently, if the derivative of its log is strictly positive, i.e. $dLog[P_i(t)/D_i(t)]/dt = P_i'(t)/P_i(t) - D_i'(t)/D_i(t) > 0$. One reason to use the latter condition instead of the former is that it helps interpretation since $dLog[P_i(t)/D_i(t)]/dt > 0$ simply states that the difference between the (instantaneous) growth rate of cumulated cases and the growth rate of cumulated tests should be positive. When cumulated cases grow at a larger rate than cumulated tests, the cumulative positivity rate goes up over time, which signals that the pandemic worsens. In other words, the derivative of the log of $P_i(t)/D_i(t)$ equals zero when the cumulative positivity rate reaches a local minimum or maximum and is about to change direction, indicating either improvement or worsening.

Another advantage of using the derivative of the log is that it does not depend on α_i and γ_i, since

$$\frac{dLog(P_i(t)/D_i(t))}{dt} = \frac{\pi_i'(t)}{\pi_i(t)} - \frac{\delta_i'(t)}{\delta_i(t)}$$

does not either, which means that it could be used to compare the dynamics of different groups that have otherwise different levels of cumulative positivity rates due to different $\alpha_i's$ and/or $\gamma_i's$. One drawback of such a measure, however, is that it is expressed in percentage points, which implies that small values do not indicate that the pandemic slows down in terms of new cases approaching zero. Rather, small values of $dLog[P_i(t)/D_i(t)]/dt$ indicate that the growth rates of cumulated cases and of cumulated tests are close to each other, which does not exclude cases in which the growth rate of cumulated cases is in fact large. This suggests that although the *sign* of the derivative of the *log* signals qualitatively whether the pandemic improves or worsens, its value is not a proper quantitative measure of how the pandemic evolves within and across groups.

One way out of this conundrum is to use instead the following scale-free (positive) elasticity:

$$\frac{dLog[P_i(t)]}{dLog[D_i(t)]} = \frac{\pi_i'(t)/\pi_i(t)}{\delta_i'(t)/\delta_i(t)}$$

as a relevant measure of both whether *and by how much* the pandemic improves or worsens. When such an elasticity is larger than one, it signals that the cumulative positivity rate is increasing over time because this happens when the growth rate of cumulated cases exceeds that of cumulated tests, i.e. when $\pi_i'(t)/\pi_i(t) > \delta_i'(t)/\delta_i(t)$. This is the same condition as above. Most importantly, such an elasticity reaches zero when the growth rate of cumulated cases also reaches zero, indicating that the pandemic has stopped producing new cases, even if temporarily so. Technically, this means that the above elasticity is a ratio-scale variable, that is, one with a non-arbitrary zero that does not depend on a particular convention about units. This property comes from the fact that, by definition, this elasticity is a ratio which expresses in relative terms the growth rate of cumulated cases compared to that of cumulated tests, in contrast with the derivative of log which is defined as the difference in growth rates. It is this property which allows us to compare in a unit-free way different groups in terms of how far from extinction of the pandemic they are.

In sum, we have shown in this appendix that the above elasticity tracks the dynamics and turning points of the cumulative positivity rate for each region/group of interest, in a way that allows scale-free comparisons between region/groups. Of particular importance is the property that this elasticity gives a measure of how far from extinction of the pandemic each region/group is, in terms of its own test dynamics. In [7, 9] we have called the discrete-time analog of the above elasticity the acceleration index, some properties of which are further delineated in the main text of this paper. Importantly, acceleration indices applied to test and case data serve as test-adjusted reproduction numbers, allowing to analyze the dynamics of infection under varying testing conditions, as shown in [9].

Supplementary Table 2: COVID-19 testing rates by year: descriptive statistics

Continent	Q1 2020	Median 2020	Q3 2020	IQR 2020	Q1 2021	Median 2021	Q3 2021	IQR 2021
African Region (AFR)	12.8221	25.5851	69.4361	56.6140	40.3993	101.1576	239.7808	199.3815
Eastern Mediterranean Region (EMR)	101.7246	203.7590	576.8400	475.1154	253.6732	513.8275	1588.5491	1334.8759
European Region (EUR)	283.1437	431.0990	542.6600	259.5162	712.1754	1448.3655	2450.4217	1738.2463
Region of the Americas (AMR)	45.2367	107.0583	204.2411	159.0044	203.7347	450.0581	775.1395	571.4049
South-East Asian Region (SEAR)	30.7790	59.4947	106.9260	76.1469	148.5700	240.6729	333.3187	184.7487
Western Pacific Region (WPR)	71.5412	126.1730	362.2386	290.6974	476.7970	781.0882	979.2198	502.4228

Supplementary Table 3: COVID-19 cases as a fraction of tests by year: descriptive statistics

Continent	Q1 2020	Median 2020	Q3 2020	IQR 2020	Q1 2021	Median 2021	Q3 2021	IQR 2021
African Region (AFR)	2.7962	6.4994	8.9556	6.1595	5.3559	8.8131	13.3546	7.9987
Eastern Mediterranean Region (EMR)	3.7916	8.4785	12.3687	8.5771	2.7617	5.3554	10.3154	7.5538
European Region (EUR)	4.3405	8.6375	14.4949	10.1544	3.7704	7.0009	9.8516	6.0811
Region of the Americas (AMR)	9.2564	18.9486	30.3114	21.0550	8.1665	14.1343	18.4500	10.2835
South-East Asian Region (SEAR)	3.7091	5.3353	11.6549	7.9458	6.4144	11.5219	13.6139	7.1995
Western Pacific Region (WPR)	0.7667	2.5053	6.2309	5.4642	1.0924	6.1498	6.7819	5.6896

Supplementary Table 4: COVID-19 case fatality rate by year: descriptive statistics

Continent	Q1 2020	Median 2020	Q3 2020	IQR 2020	Q1 2021	Median 2021	Q3 2021	IQR 2021
African Region (AFR)	0.9006	1.7024	2.0525	1.1519	1.2355	1.4681	2.5925	1.3570
Eastern Mediterranean Region (EMR)	0.5621	1.6895	2.1084	1.5463	0.5735	1.0858	1.4737	0.9002
European Region (EUR)	1.0726	1.7736	2.6181	1.5455	0.6259	0.9921	1.7793	1.1533
Region of the Americas (AMR)	1.8153	2.6475	3.5237	1.7084	1.4065	1.8829	2.7212	1.3147
South-East Asian Region (SEAR)	0.5932	1.0153	1.3501	0.7570	1.0698	1.4538	1.8284	0.7586
Western Pacific Region (WPR)	1.4093	1.4785	1.8195	0.4102	0.6202	1.0019	1.4746	0.8544

Supplementary Table 5: Kendall correlation coefficient between pairs of infection acceleration indices and p-values

	AMR	EUR	SEAR	EMR	WPR
AFR	0.3218 (.0000)**	0.2139 (.9988)	0.0925 (.0943)	0.0700 (.1587)	-0.0501 (.7627)
AMR		0.1859 (.0039)**	0.0434 (.2685)	0.1560 (.0129)	0.2948 (.0000)**
EUR			-0.1598 (.9883)	-0.0787 (.9883)	-0.0139 (.5789)
SEAR				-0.0139 (.0000)**	0.1996 (.0022)
EMR					0.1844 (.0042)**

Supplementary Note 1: Factors affecting COVID-19 testing rates in 2020 and 2021

This Supplementary Note aims to identify some factors that influenced COVID-19 testing volumes across different countries and continents during 2020 and 2021. Specifically, we analyze how key variables such as GDP per capita, the CEO Health Index, the WHO Health Index, the Democracy Index impacted the number of tests conducted per 1,000 inhabitants, as shown in Supplementary Figure 1.

Correlation coefficients for each variable with testing volumes, reported in Supplementary Figure 2 are indicative of the relative importance of these factors in driving testing efforts, particularly in the early stages of the pandemic versus later periods, i.e. 2020 vs 2021 in our sample. The analysis was visualized using graphs to illustrate trends and shifts in the strength of these correlations over time. Our initial hypothesis was that wealthier nations, as indicated by higher GDP per capita, would show a stronger testing response, particularly in 2020 when the pandemic's uncertainty was at its peak. This analysis provides a foundation for understanding the broader socio-economic factors that influenced public health responses globally.

The results of the correlation analysis presented in Supplementary Figure 2 confirmed that GDP per capita was a significant determinant of testing volumes in 2020, with a correlation coefficient of 0.67, indicating that wealthier countries were more likely to conduct widespread testing. In this context, GDP per capita can be interpreted as an indicator of a country's preparedness for the epidemic. Nations with higher GDP per capita are likely to possess the financial, political, and logistical resources necessary to mount an effective response to the crisis. These resources enable them to implement widespread testing, manage healthcare infrastructure, and deploy public health initiatives more rapidly and efficiently compared to countries with lower GDP per capita.

However, this influence waned in 2021, with the correlation dropping to 0.36. A similar pattern was observed for the CEO Health Index (from 0.45 in 2020 to 0.35 in 2021) and the Democracy Index (from 0.54 to 0.38), suggesting that as the pandemic progressed, these macroeconomic factors became less predictive of testing behavior.

By 2021, macroeconomic indicators have significantly diminished in their ability to explain testing capacities. This shift is likely due to the substantial variance in the number of tests per 1,000 inhabitants across countries, which is no longer adequately captured by these broad indicators. The only variable that remains significant in 2021 is the European region. This suggests that testing capacity in 2021 is influenced by other factors beyond traditional macroeconomic variables. For instance, countries with nearly equivalent GDPs, such as France and the United Arab Emirates, display vastly different testing rates per 1,000 inhabitants-2269.419 in France versus 8982.786 in the UAE-indicating that other elements, possibly including government policy, public health infrastructure, or regional strategies, are driving testing efforts.

Linear regression analysis, reported in Supplementary Table 6, further reinforced these findings, showing that GDP per capita had a significant positive impact on testing volumes in 2020 ($p = 0.0061$), and that democracy index as well ($p = 0.0292$), but no variable, including GDP per capita, was significant in 2021. Only the EUR indicator shows up as significant in 2021 ($p = 0.0139$), indicating the Europe tested significantly more then. This shift implies that other, possibly more complex factors influenced testing rates as the pandemic evolved, and that the initial economic capacity-driven testing strategies became less relevant. The adjusted R-squared values, which dropped from 0.47 in 2020 to 0.13 in 2021, highlight the increasing unpredictability and complexity of the determinants of testing as the global situation evolved. These findings suggest that the determinants of pandemic response strategies, including testing, shifted over time, reflecting changes in both the pandemic's trajectory and global policy responses.

Supplementary Figure 1: GDP per capita, democracy index and health index per countries grouped in regions against population testing rates in 2020 and 2021

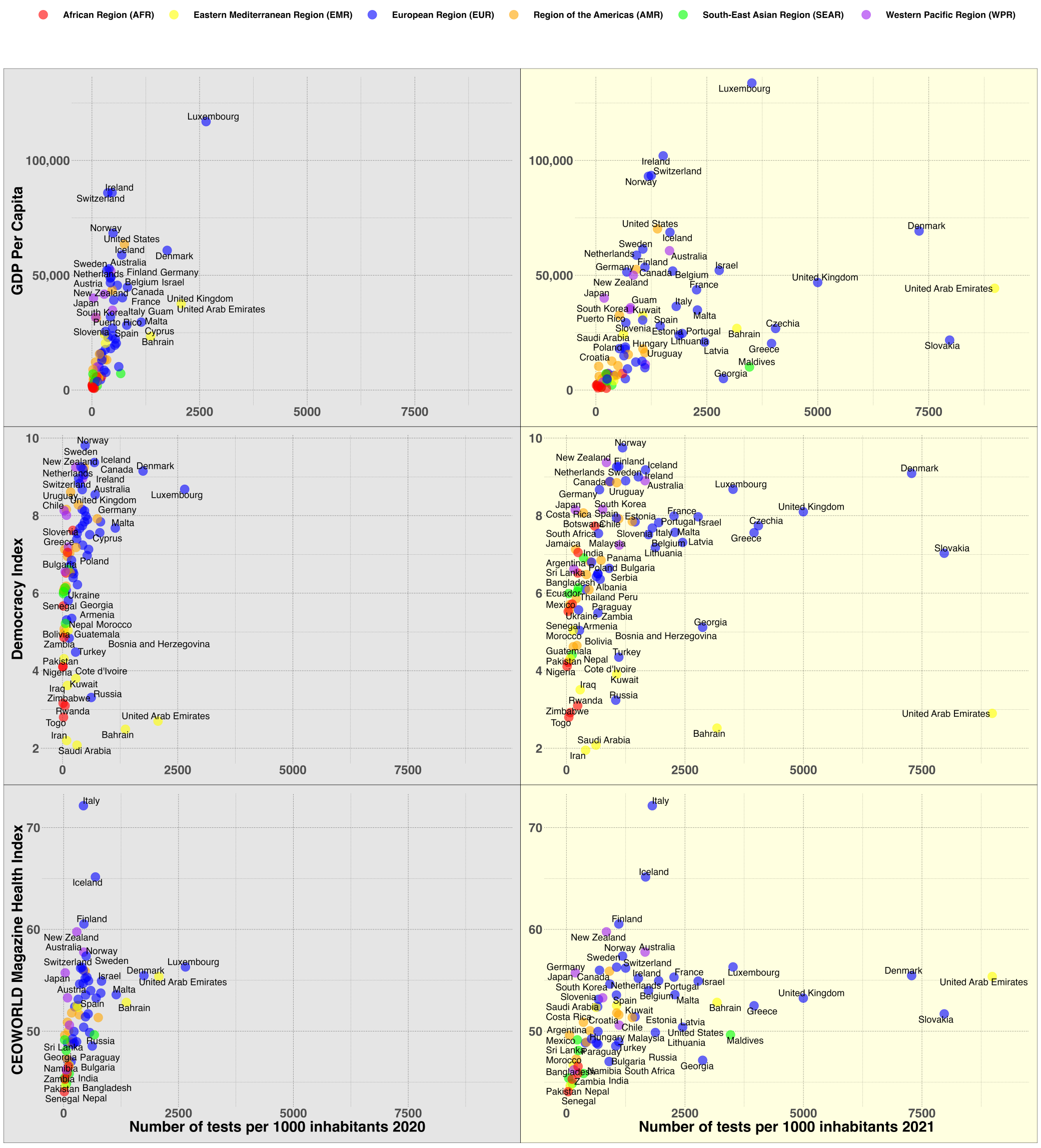

Supplementary Figure 2: Correlation between testing rates and country specific characteristics in 2020 and 2021

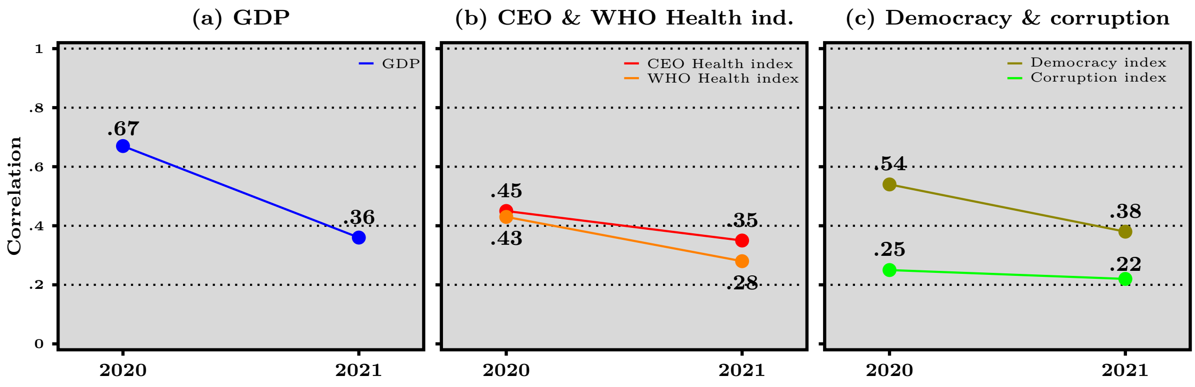

Supplementary Table 6: Linear regression results for population testing rates as explained variable in 2020 and 2021 (Reported p-values are based on two-sided Student's t-tests)

	2020		2021	
**Variable**	**Coefficient**	**p-value**	**Coefficient**	**p-value**
Intercept	0.4537	0.4632	-1.4649	0.6174
ContinentAfrican Region (AFR)	0.0137	0.8489	0.0577	0.7564
ContinentEastern Mediterranean Region (EMR)	0.0526	0.7956	1.3168	0.2001
ContinentEuropean Region (EUR)	0.1177	0.1358	1.7010	0.0139*
ContinentRegion of the Americas (AMR)	0.0955	0.2425	0.1467	0.6328
ContinentWestern Pacific Region (WPR)	-0.1099	0.3097	0.0314	0.9474
gdp_per_capita	0.0000161	0.0061**	0.0000157	0.2442
CEO2023	0.0062	0.6342	0.0513	0.4974
democracy_index	-0.1232	0.0292*	-0.1321	0.6285
**Adjusted R-squared**	0.4733		0.1344	

Supplementary Figure 3: Infection and mortality accelerations in 2020 and 2021 for 139 countries (excluding China)

The Figure presents both raw and estimated acceleration indices of infection and mortality dynamics for 139 countries (excluding China) during 2020 and 2021. Estimations are carried out using locally-weighted polynomial regressions (LOESS). Blue (resp. red) shaded areas indicate 95% confidence of intervals of infection (resp. mortality) estimated indices. Each year is divided into quarters to facilitate reading. Results show that our findings are robust despite incomplete data and delays in processing and reporting for included countries. secondary database

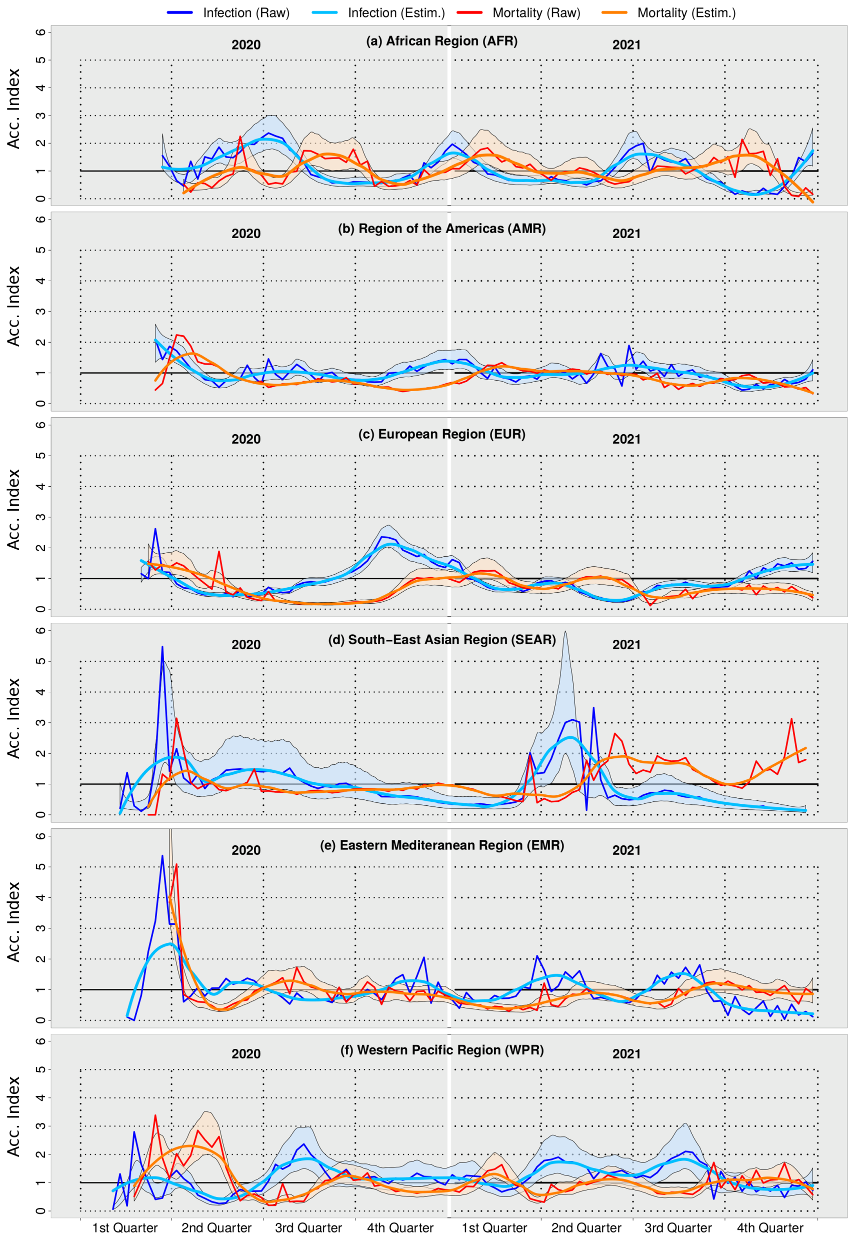

Supplementary Figure 4: COVID-19 acceleration indices for infection and mortality in Western Pacific Region for 140 countries (including China), for 2020 and 2021

The Figure illustrates the acceleration indices of infection and mortality dynamics for the Western Pacific Region, including China's reported cumulative tests, cases, and deaths. Estimations are carried out using locally-weighted polynomial regressions (LOESS). Blue (resp. red) shaded areas indicate 95% confidence of intervals of infection (resp. mortality) estimated indices. Each year is divided into quarters to facilitate reading. The data reveals a dramatic upward shift in infection dynamics starting in the third quarter of 2020, with a peak estimated acceleration index of 15.9. However, this increase in infection does not appear to have immediate consequences on mortality dynamics, rather surprisingly. Understanding these results likely requires a more in-depth analysis, which is beyond the scope of this paper.

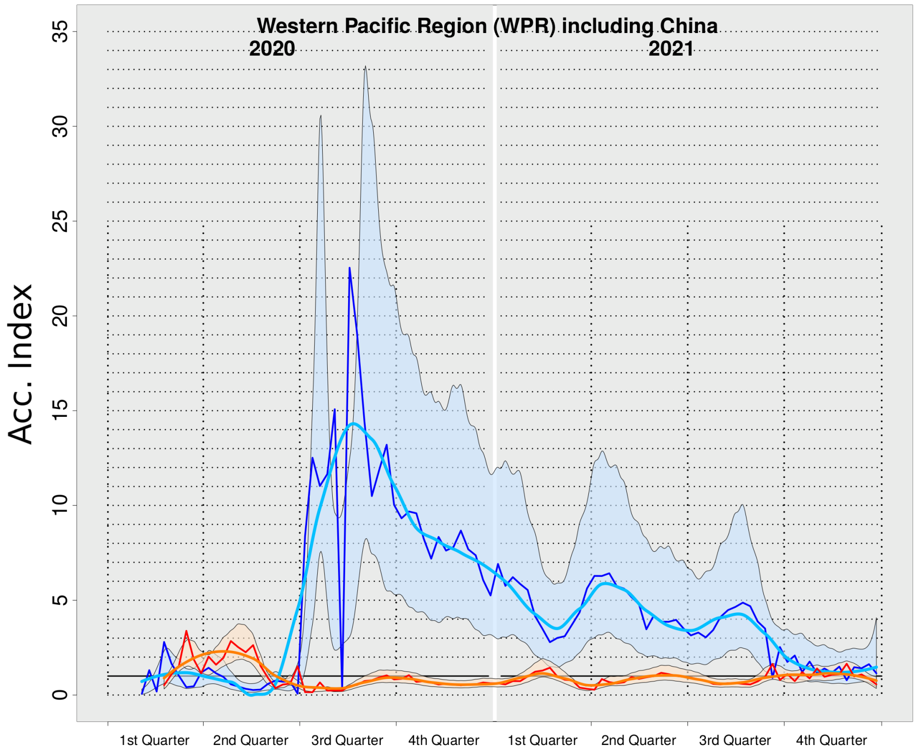

Supplement: Supplementary file 2 — Supplementary information [file 43856_2025_1343_MOESM2_ESM.pdf]
